# Patients’ experiences and preferences for primary care delivery: a focus group analysis

**DOI:** 10.1017/S1463423619000422

**Published:** 2019-06-23

**Authors:** Patrícia Norwood, Isabel Correia, Paula Veiga, Verity Watson

**Affiliations:** 1 Health Economics Research Unit, University of Aberdeen, Polwarth Building, Aberdeen, UK; 2 JusGov, School of Economics and Management, University of Minho, Braga, Portugal

**Keywords:** patient’s preferences, Portugal, primary health care

## Abstract

**Background:**

In 2005, the Portuguese government launched a primary care (PC) reform. After a promising start, the reform is still incomplete and has been compromised by low investment. The incomplete nature of the reforms has resulted in the coexistence of different models of care delivery and heterogeneity in resource allocation and performance. PC has been extensively evaluated, but little is known about the patients’ views and preferences regarding PC and the ongoing reform.

**Aim:**

This study aims to examine patients’ experiences of and preferences for PC in Portugal and to explore their experience of the recent reforms.

**Methods:**

A qualitative study was undertaken which collected data from eight focus groups in the city of Braga, Portugal. Participants were recruited with the collaboration of eight local institutions. Focus groups’ discussions focused on patients’ experiences of and preferences for PC as well as their views on the reforms. Audio recordings were transcribed and analysed using an inductive thematic content analysis.

**Findings:**

The majority of participants perceived that the reform was positive. However, the improvements achieved by the reform were insufficient to lead to most participants having a positive experience of PC delivery in Portugal. Participants’ satisfaction/dissatisfaction with primary care was strongly associated with interpersonal relations and communication with doctors. Participants valued continuity of care, but felt the levels of responsiveness, flexibility and coordination in the current system were still unsatisfactory. Access and waiting times were seen as challenging and led participants to seek PC from emergency departments and private doctors.

**Policy Implications:**

The perception of increased inequity and the lack of effective choice undermined the social acceptability of the reform. Policies aimed at improving doctor–patient communication and continuity of care, as well as choice, may therefore lead to better satisfaction and more efficient use of health care settings.

## Introduction

The Portuguese health care system is characterised by three coexisting, overlapping systems: the universal National Health Service (NHS); special public and private, professional insurance schemes (health subsystems that cover 25% of the population) and private voluntary health insurance (covering 10–20% of the population) (Barros *et al*., 2011). The NHS is defined as universal, nearly free at the point of use, and mainly funded by general taxation.

The Portuguese NHS is organised as three levels: primary, secondary (mainly hospital care) and tertiary care (very specialised care, continuity care and palliative care). In 2013, Primary Care (PC) accounted for approximately 13% of NHS expenditures (INE, 2017). Access to PC is constrained by a user charge; however, more than 50% of the population (Administração Central do Sistema de Saúde (ACSS), n.d.) (including children, lower-income families, patients with serious disabilities and many others) are exempt from these charges (Observatório Português de Políticas de Saúde (OPSS), 2013). Furthermore, patients pay co-payments towards the costs of prescribed drugs and examinations.

The PC system has two functions. PC should provide relational and longitudinal continuity of care and to act as a gatekeeper to non-urgent secondary care. Individuals (and, if possible, families) are expected to register with a family doctor (FD) who works in a PC unit. Individuals are legally entitled to choose the PC unit and FD. In reality, individuals have limited choice because there is a shortage of doctors (Simões *et al*., 2017). In addition, individuals do not have easy access to information about the choices available to them or, until December 2017, information about the performance of FDs or the PC units in which they are based.

The Portuguese primary health care reform was launched in 2005 and has deeply changed PC. These reforms have been supply-led and have reshaped the organisational role of general practitioners (GPs), their clinical and management activities and incentives. In Portugal, there are two types of PC unit: Family Health Units (FHUs), and Personalized Health Care Units (PHCUs). The main focus of the reform was to create and expand the coverage of FHUs. These are autonomous units of self-selected GPs, nurses, managers and other professionals within a flexible organisational structure that has autonomy. FHUs’ remuneration is based on a combination of salaries, capitation and pay for performance (Simões *et al*., 2017). Virtually all patients in an FHU are assigned to an FD. In contrast, PHCUs comprise health professionals who have not joined an FHU. They are characterised by poor team dynamics and a lower autonomy (Observatório Português dos Sistemas de Saúde, 2012). PHCUs serve patients not registered with an FHU. There are significant disparities in the quality of care and outcomes between FHUs and PHCUs. FHUs have a lower numbers of patients per FD, provide a higher level of service (Observatório Português dos Sistemas de Saúde, 2012) and have high(er) patient satisfaction when compared with PHCUs (Ferreira and Patricia, 2009; Mendes *et al*., 2013). In line with international experiences (Chapman *et al*., 2004), the Portuguese PC reform also aimed to change the PC workforce skill mix delivering PC by expanding the roles of healthcare professionals, such as nurses.

Over a decade after the reform was launched it is still not fully implemented (Ministério da Saúde, 2017). The number of FHUs has steadily increased, but not by enough to adequately cover the population (Ministério da Saúde, 2017). It was expected that all PHCUs would evolve into FHUs, but the process has been slow and is still incomplete. At the time of data collection, in 2013, more than half of the Portuguese population was not registered with an FD working in an FHU and was therefore registered with PHCUs. Furthermore, 13% of the population were not registered with an FD at all. By 2017, these percentages have decreased to 41.9% and 7.3%, respectively. (Ministério da Saúde, 2017). Despite being considered one of the most successful reforms of the country’s public services (Biscaia and Heleno, 2017), the PC reforms have not met their goals and terms of equity of access and use. However, little is known about patients’ experiences of the reformed system, and how they are affected by the incomplete nature of the reforms. To do so, we carried out a descriptive qualitative study using focus groups to examine patients’ views and preferences for PC services and to explore their experiences of the recent reform.

## Methods

We used a series of focus groups to explore patients’ experiences of PC in Portugal. Our overarching aim was to obtain rich and detailed information about patients’ experience of PC in Portugal. Within this, we aimed to understand how patients’ experiences were affected by: the organisation of PC services; the type of PC unit they were part of; and the reforms. We realised that patients might not be knowledgeable about the reforms and their objectives; therefore, we aimed to understand how patients experienced key aspects of PC that aligned with the reforms, specifically, access, continuity of care and cost. We chose to collect data using focus groups because it allowed us to understand how people think and reason about PC and to understand why people think the way they do (Krueger and Casey, 2000). While FHU and PHCU share organisational features, we were aware that there is diversity within different units, and focus group interactions would allow us to learn about this by hearing participants compare and contrast their experiences. Therefore, the interaction between participants was important to our research aims by encouraging participants to explore and clarify their views (Kitzinger, 1995) and provide us with access to their shared experiences, values and understandings (Owen-Smith and Coast, 2017).

The focus groups for this study were held in the municipality of Braga, Portugal. Braga is a mainly urban municipality (INE, 2009) located in the north of Portugal. The area has a wide diversity in the way in which PC units are organised, which meant that focus group participants were likely to have experienced different organisational types.

Participants were recruited with the collaboration of eight institutions: University of Minho, two private firms, three cultural associations, two local government organisations (‘Juntas de Freguesia’). The use of organisations to recruit focus group participants is one of the strategies suggested in the literature when the topic is sensitive and researchers face difficulties in recruiting (MacDougall and Fudge, 2001). The organisations were selected purposively to ensure variation in areas and participants’ age, socio-economic background, access to PC and health care needs. One group consisted of pregnant women and another of senior individuals. These groups were targeted because we believed that they would have specific PC needs that might potentially result different preferences from the rest of the population.

The organisations received an email that explained the purpose and methods of the study. Participants who were adults over 18 years old were recruited by the organisations using two additional criteria: they would have something to say on the topic (Krueger and Casey, 2000) and they would be comfortable talking to the facilitators and to each other (Richardson and Rabiie, 2001). Data collection was stopped when we reached saturation and no new themes were emerging in the focus groups.

Eight focus groups (69 participants) were held between March and July of 2013. They were held in facilities provided by the organisations, on the day and time previously agreed. All the facilities provided were comfortable, and refreshments were available to participants. On average, the focus groups lasted one hour, but durations of the focus groups varied from 35 to 105 minutes (Table 1).

**Table 1.** Characterisation of the focus groups

	Place	Duration (minutes)	No. of participants	Area	Participants
FG1	Local government	60	6	Urban area	Members of the community
FG2	Firm (Preparation for birth delivery)	35	9	Urban area	Pregnant women and their partners
FG3	Local government	45	10	Predominantly urban area	Members of the local community
FG4	Senior Association	90	10	Urban area	Seniors
FG5	Firm (Software development)	60	5	Urban area	Firm employees
FG6	Social and Parochial Centre	70	8	Predominantly urban area	Members of the Local Community
FG7	University of Minho	105	13	Urban area	Employees; mainly public servants
FG8	Local government	60	8	Moderately urban area	Members of the Local Community

The focus groups had between 5 and 13 participants. We aimed to have sufficient participants to be able to include people with a range of viewpoints (Tang and Davis, 1995) whilst at the same time ‘allowing participants to interact’ (Corbetta, 2003). In some focus groups, participants had similar experiences and this allowed us to understand about people’s shared experiences, while other focus groups brought together people with diverse experiences which allowed us to explore different perspectives within a group setting (Kitzinger, 1995).

Two members of the research team were present in all focus groups (Veiga and Correia). In each group, one team member assumed the role of the main facilitator, while the other took notes. The facilitator used a semi-structured topic guide, informed by the literature review and discussions across the research team (Owen-Smith and Coast, 2017). Participants were asked about their experiences with public PC services, what was important to them, their satisfaction with the services and the reforms (Box 1). The facilitator’s interference with the discussion was kept to a minimum and was focused in guiding the discussion. Discussions were audio-recorded with the consent of the participants.

**Box 1.**
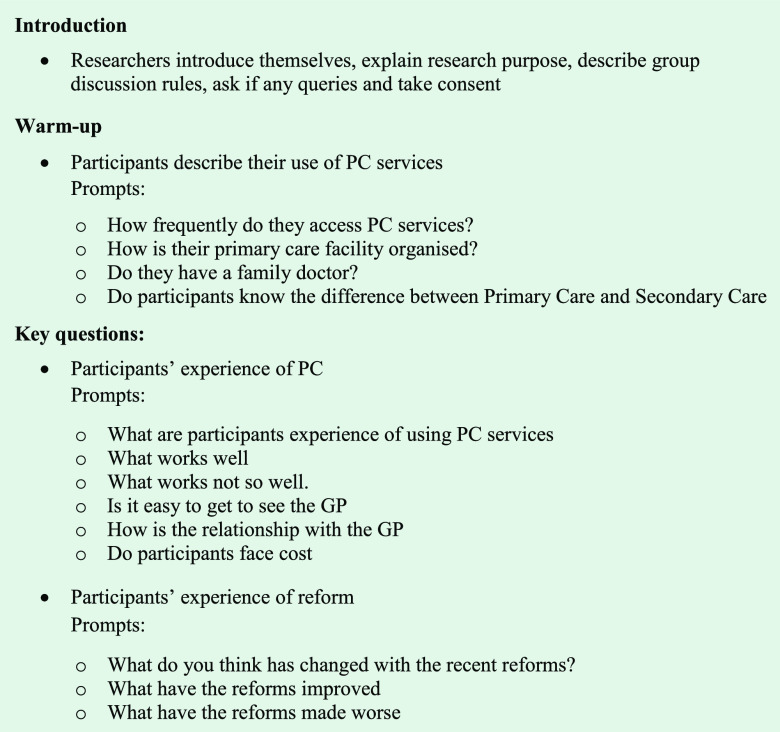
Focus-Group Guide Questions

Participants provided written informed consent before the start of the discussion. All procedures followed were in accordance with the ethical standards of the University of Minho at that time and Helsinki Declaration of 1975, as revised in 1983. Participants were also asked to complete a brief questionnaire designed to collect information regarding socio-demographic and health characteristics, PC service use and satisfaction with public PC providers. Confidentiality was emphasised.

## Analysis

The focus group discussions were transcribed verbatim. The recordings were listened to several times to ensure the accuracy of the transcription. Qualitative data analysis involved the reading and rereading of the transcripts in Portuguese by the two team members who were in all focus groups and by one other member of the research team who did not take part in focus group discussions. These three researchers coded the transcripts independently. This meant each focus group transcript was coded by three people. An inductive thematic approach was used (Braun and Clarke, 2006). The coding process was informed by the literature on PC services and organisation (Donabedian, 1988; Wensing *et al*., 1998a; 1998b; Grol *et al*., 2002; Jung *et al*., 2003; Coulter, 2005; Cheraghi-Sohi *et al*., 2006; WHO/Europe, 2010).

Through the coding process, statements that were relevant to the research aims were identified and initial codes were generated. These codes identified features of the data that the researchers considered pertinent to the research question. The second stage involved combining different codes that were similar or addressed the same issue. All initial codes relevant to the research aims were grouped and further developed into multiple dimensions. The third step involved combining each member’s independent analyses, based on team consensus. Anonymous quotes from these transcripts, translated into English, illustrate the main themes in the results section.

## Results

Table 2 summarises the socio-demographic characteristics of the participants based on responses to the short questionnaire distributed in the focus groups. The majority of participants were female (69%); 44% of participants were under 50 years old. The conditions for a continuous doctor–patient relationship were relatively good: about 81.1% of the participants reported that they have an FD in the public system and nearly 28% of participants have a private PC provider that they consult regularly. Almost 56% of patients reported being with their doctor (either public or private) for more than five years.

**Table 2.** Participants’ characteristics

Characteristics	**%**
Female	69.1
Age:	
▪ 20–34	14.7
▪ 35–64	61.8
▪ 65 or more	23.5
Have a regular provider (either private or public) ▪ public provider	89.7 85.3
How long with the current provider:	
▪ Less than one year	11.9
▪ One–five years	30.5
▪ More than six years	57.6
Have a health subsystem or private insurance	53.8

Note: The number of respondents is not the same for all the questions. Only 59 respondents completed the time with current provider.

The majority of the participants were familiar with the basic characteristics of public PC organisations, but they did not always recognised public PC services or boundaries between PC and other types of care such as secondary or hospital care. Therefore, in the focus group, the facilitator had to define PC services and provide several examples to illustrate the scope of public PC services in the warm-up stage of the focus groups.

The final theme structure closely followed Donabedian’s classic division (Donabedian, 1988): (1) structure, (2) process and (3) outcomes of primary health care. We examined the frequency with which each theme was mentioned in the focus groups (Krueger and Casey, 2000) and found the main themes identified were related to the process of PC delivery. This is the overarching theme that this paper focuses on. It should be noted that cost of care was not one of the emergent themes – it was rarely mentioned by participants. This was an unexpected result because features of the reform were the introduction of user charges. A possible explanation for this being rarely mentioned is that the exemption system to the user charges is extensive and those who have to pay user charges do not find them an issue of concern.

### Access and waiting time

Participants identified access and waiting times as problematic within PC. Access was a particular issue, with participants often mentioning an inability to get an appointment with their FD quickly. Across the focus groups, participants reported that waiting times for appointments with GPs could extend to several months. For example, in focus group 1 (FG1), one respondent stated:

“*When I need an appointment I will see whatever doctor is available but I should be seen by my family doctor shouldn’t I? I think that [waiting for] 2/3 months is too long!*”

In addition to long waiting times for an appointment, participants reported significant delays while waiting in the practice for their appointment. Patients could not rely on fitting the appointment in with their schedule. For example, a participant in focus group 3 (FG3) stated:

“*We arrive at 8am, but he [the doctor] only arrives at 11am. We think this is a lack of respect*.”

Participants valued flexible access to PC appointments and PC units with long opening hours and flexible booking. For example, a participant in focus group 4 (FG4) stated:

*“… my doctor starts the appointments at 8am so she can see first the patients who work.”*

In our urban context, proximity to the PC unit was not important to participants. Nonetheless, access was discussed in terms of the lack of public transport to the location, lack of parking nearby and accessibility of the location to people with physical limitations. For example, participants in focus groups 5 and 8 (FG5 and FG8) stated:

“*One thing that is missing in my Health Care Centre is a parking lot. This is a problem to me, as my [premature] baby is very fragile (…) someone has to drive me because there is no parking in or near the FHU.”*

*“Unfortunately there’s no public transportation [to the health centre]. We have to go by private transport.”*

Almost 38% of our participants are exempt of user fees on the basis of low income or chronic disease. Therefore, unsurprisingly, user fees and the recent increase of user fees were seldom perceived as a barrier to PC access. Nevertheless, there was a consensus among the groups that financial difficulties should not prevent anyone from accessing care; however, income related co-payments were tolerated. While some participants believed in a free at the point of use NHS, the majority appeared to agree with income-related co-payments with (a broader) exemption system. For example, two participants in focus group 5 (FG5) stated support for means-tested co-payments that were not a barrier to access:

*“There are the user fees, but maybe they could create some strata so that we pay according to our financial situation. However, access should be for everyone.”*

*“I think that we should pay for the service provided, but it has to be somehow fair.”*

### Patient–doctor relationship

Participants greatly valued the patient–doctor relationship. A positive patient–doctor relationship was multifaceted and included evidence that the GP listens, invests time in identifying and solving problems, puts patients first and treats the patient with respect and ‘humanity’. A non-empathic GP was perceived as a signal of lack of interest in the patients and leads to feelings of insecurity regarding quality of care and to potential psychological harm.

Participants would like to wait for less time and have longer consultations with their GP. Nonetheless, participants recognised that GPs who have many registered patients have less time to treat each patient and that this detrimentally affects the quality of care that patients receive. For example, one participant in focus group 6 (FG6) stated:

*“[It is necessary] to hire more doctors so each doctor has less patients because it’s impossible to treat [equally] well 10 or 100 [patients], isn’t it? It’s different amounts of time for [each] person. I think we lack enough doctors.”*

Positive patient–doctor interactions were more often reported by participants who were registered with FHUs. Patients seemed to approve of the creation of FHUs. In FG6, one participant stated:

*“…[FHUs] are very well organized: one doctor, one nurse (…) for each patient and their family (…)”*

Nonetheless, often participants expressed concerns with the reform increasing the already persistent inequities in care, as the result of three-tiered model of delivering care (patients with FD in an FHU, patients with FD in a PCHU and having a ‘good’ or a ‘bad’ FD) and the dichotomy of public/private PC.

### Continuity of care

Participants valued continuity of care and having a doctor who knew their and their family’s medical history. For example, a participant in FG6 stated that continuity of care was one of the aspects of the reforms that had most improved care:

“*They [FDs] get to know the families better, the patients and I think that is, it is one of the best things that has improved lately…*”

Some participants recognised the advantages of continuity of care but were, nevertheless, sceptical about the rigidity of the system, which comes from the perception that patients with an FD only ever receive care from that GP, whereas patients without an FD can receive care from any GP in a practice. This means that patients’ lack of access to a second opinion. This combines with a lack of effective freedom to choose the GP to leave participants ‘stuck’ with a GP. For many, the discussion around the possibility of choice seemed to be surprising. Some participants reported trying or wanting to change to a different FD. These patients were unable to change FD or were advised against trying because of shortages of FD. For instance, a participant in FG1 reported being unable to change because there was no available FD:

*“Can I switch my family doctor? Unfortunately, I cannot. I have tried, but I could not because there are no available doctors – I wish I could*.”

Patients with a good patient–doctor relationship wanted continuity and were even willing to travel to distant GP practices in order to keep the same FD. However, participants recognised the pitfall of being registered with an FD with whom they have a negative patient–doctor relationship or an FD with whom it is difficult to arrange an appointment. For instance, a participant in FG1 believed that continuity of care might bring better health outcomes, but still stated a resistance to have an FD for fear of a bad relationship:

*“[I’ve never wanted a FD] because if it’s always the same doctor, if I get a bad professional then things won’t work out. But on the clinical side, maybe it would be best to have a FD that is always the one to see me.”*

Some participants would prefer a more effective choice in PC, including choice among alternative models of PC delivery. A participant in focus group 7 (FG7) stated:

*“I think there should be a choice between having just one family doctor, and being constrained to seeing just that doctor, or going to a health care centre and seeing one doctor on duty.”*

Participants praised informational continuity of care and expected their health care providers to keep medical records updated and available. Overall, they trusted the information system within their PC unit and believed clinical records make it easy to be treated by any doctor. Some participants perceived informational continuity was a close substitute for, or even better than relational continuity. A participant in FG7 stated:

*“… I don’t care about who is the doctor, as long as he has my file in front of him.”*

Management continuity within the PC unit is perceived as an important improvement of FHUs: quite often participants reported that their FHU contacted them, either by post or by phone, to reschedule/cancel an appointment or to remind them about an appointment. It made them feel like someone cared about their health. For instance, a participant in FG5 said:

*“I wasn’t expecting to receive a letter at home saying: you have an appointment at “x” o’clock with the doctor. I thought they did not care.”*

### Comprehensiveness of PC

In general, participants expressed few expectations for specific services to be provided by PC, other than curative treatment, referral to secondary care and the prescription of medication and tests. The discussion about the lack of comprehensiveness and technology in PC was only relevant in the focus group of pregnant women. In FG2, one participant stated:

*“Health Care Centres should provide information about what (they) can provide.”*

Recent austerity was perceived to have reduced the volume of tests that participants received. This was perceived negatively and participants never questioned whether previous tests they had received were necessary. For instance, one participant in FG1 suggested that his GP’s recommendation regarding testing frequency was based on budget restrictions:

*“[Regarding the cost cuts] for the last seven years I used to do lab tests every six months, and since last year [2012] I started to do them once a year. Something happened, and it was that [cost control], right?”*

### Appropriateness and Coordination in care

Participants noted persistent inefficiencies across the public PC sector, due to lack of organisation and coordination. Participants reported that patients were particularly vulnerable when they are making a transition between care settings and services. Participants describe the system as being very fragmented and they did not perceive that there was a credible patient pathway.

The first hurdle that people report facing is the referral from PC to the secondary care. This was particularly difficult for participants without an FD. Prompt and adequate referral to hospital outpatient care is highly valued and is perceived as the doctor caring, but also having technical competence. For example, in FG1 and FG3 participants stated:

*“She [the FD] did not refer my dad to the hospital. She said he was only stressed. My father had a lung cancer and died aged 49.”*

*“[The FD] only refers [to the hospital] in the limit, only in the limit.”*

A second hurdle that people reported is the long waiting time after being referred before being able to access to specialised care. Examples include statements from participants in FG1 and FG7:

*“I have hepatitis C. I waited 4 years for an hospital appointment.”*

*“… after referral, it took 2 years till the hospital appointment.”*

Participants perceived that the long wait was caused not only by lack of organisation or coordination within the health care system, but also as a lack of power of the GP/FD. Participants also identified a lack of communication and information transfer between providers as a barrier to accessing secondary care. Participants perceived that better information technology would improve the system. Two participants in FG8 exemplified these concerns when they said:

*“I have noticed many times that the FD does not like to receive orders or opinions from hospital doctors…”*

*“There should have some coordination between social security, domiciliary care, health care centre, hospital… everything would run more smoothly.”*

Most of the participants recognised the institutional role of their FD as a gatekeeper for hospital care and identified the idealised pathway of patients along the NHS. Nonetheless, long waiting times for an appointment combined with uncertainty about whether the patient will see the doctor after a long wait contributed to patients’ use of hospital emergency departments and private specialist care (in particular, individuals covered by a subsystem). When patients needed urgent care, they preferred to get care in an emergency department setting, even if they would have to wait a long time. For example, participants in FG3 and FG8 stated:

*“When I need it [urgent health care], I rather go to the hospital because I know I will be seen. If I go to a [primary care unit] I can wait for a long time [in the waiting room] and still not be seen and then I will end up having to go to the hospital anyway.”*

*“… [people] go straight to the emergency room [hospital] They can wait, two, three, four hours, but [at least] they can be seen by a doctor.”*

### Public versus private care

Participants perceived public PC as inferior to private PC. This may partly explain the prevalence of dual use of private and public care by participants. For instance, a participant in FG5 stated explicitly that individuals who can afford to will always seek private care:

*“There is the perception, accepted until very recently; that only the poor people will use public primary care, those who can afford it will run to the private.”*

In public PC units, GPs were seen as rushing though appointments and not having sufficient time to spend with any one patient. For example, a participant in FG3 when discussing the patient–doctor relationship with her FD stated:

*“The problem is that he [the GP] is always saying ‘next, next, next’, is always rushing because he has many patients and he doesn’t meet the schedule.”*

A frequently mentioned reason for using public PC services seems to be to obtain an NHS prescription. Participants do not expect their FDs to object to providing them with a prescription, including prescriptions for examinations recommended by their private doctors. One participant in FG3 said:

*“I like my doctor. He [the doctor] is really good at prescribing (…) drugs, exams and so on.”*

This complicity in which public GPs replicate decisions taken by private doctors was often reported in our focus groups. For example, in FG2, this participant sought private care because she perceived it to be safer, but choose the public PC to bear the cost of tests and prescriptions:

*“I am being seen in both the private and public system. I go to the public for the prescriptions and tests and to the private because it is safer, isn’t? I want to be seen by someone who really knows.”*

A participant in the focus group with pregnant women described this process as something that was very usual among pregnant women. In particular, there was a view that prescribing was the only role for a FD in prenatal care:

*“I went to the [public] appointment just to do a check-up or exams; and I do believe that’s the main reason [pregnant] women go to the GP, for what I can see, because they can really just prescribe you the exams and you can have like a referral?”*

## Discussion and Conclusions

The purpose of this study was to use focus group discussions to obtain detailed insight into patients’ experiences of PC services in Portugal and to explore how they have experienced the effects of PC reform. We found that patients expressed some dissatisfaction with PC services in spite of the generalised perception of improvements introduced by the recent reforms. Participants who were registered with an FHU expressed, in general, a greater enthusiasm with the reform, but overall patients want a faster and easier access to care, compassionate doctors and better responsiveness and coordination in health care system. Continuity is valued but not at the expense of freedom of choice of FD. These conclusions have important implications for health policy and practice.

Several of our results are in line with existing literature. Participants most often discussed satisfaction/dissatisfaction with PC when they talked about interpersonal relations and communication with providers, mainly with GPs (Wensing *et al*., 1998a; Grol *et al*., 2002; Osvaldo *et al*., 2007; Wong *et al*., 2008). Policies aimed at improving doctor–patient communication would thus be likely to lead to improved levels of satisfaction and appropriate use of PC. Despite the aims of the reform, patients’ experiences are still GP centred and relationships with other health care professionals seldom emerged spontaneously in the discussion.

Improved access and waiting times was one of the main aims of PC reform, but participants were still experiencing issues with both. Participants discussed issues related to access more often than any other theme. Portugal has a relatively high number of doctors per person compared to OECD countries (OECD, 2017). However, the system’s rigidity and access problems mean there is still a shortage of available GPs. We found that waiting times are a barrier to access and this appears to lead to lower satisfaction and inappropriate use of emergency departments for medical cases that could be managed in PC, thus increasing healthcare costs.

In common with previous studies, participants seldom mentioned medical outcomes. This reflects the difficulty of associating the provision of PC with patients’ outcomes (Chapple *et al*., 2002). Participants assumed that doctors have technical competence, except when they have experienced any medical error or negligence. This result is also consistent with previous studies (Markham *et al*., 1999; Fung *et al*., 2005). Other aspects of the PC process identified as important in the literature such as patient involvement, clinical information, privacy, safety, comfort, whole care, nursing role and access to second opinion (Wensing *et al*., 1998a; 1998b; Grol *et al*., 2002; Jung *et al*., 2003; Coulter, 2005; Cheraghi-Sohi *et al*., 2006) were seldom discussed in our focus groups. This may be attributable to cultural differences, in line with (Grol *et al*., 2002) but nonetheless, the focus group discussions suggest that overall participants lack experience and/or expectations of these aspects of PC.

A curative view of PC is still prevalent, participants preferred medical technology and specialised care, rather than preventive care and a holistic approach. There was a general feeling among participants that PC provided by hospitals or private doctors is better than public PC. This lack of ‘credibility’ of public PC contributes to excess hospital and specialist care use. This is consistent with previous studies (Scott *et al*., 2003). Our findings suggest that initiatives to reduce hospital demand and overuse of care should include awareness of PC matter. Public cost saving in the health care system can be achieved by treating patients in PC rather than in hospital emergency care departments.

We found that FHUs appear to be the most successful and well-known part of the reform. The focus group discussions showed that, for those enrolled in an FHU, the reforms had led to improved access to care, better patient–doctor relationships and relational continuity. Participants associated continuity with better outcomes. Nonetheless, patients differed in the importance they placed on different types of continuity. The same result is reported elsewhere (Schers *et al*., 2002; Hjelmgren and Anell, 2007; Turner *et al*., 2007). Flexibility and choice was an important issue in the discussion. Participants want a more flexible system that puts patients first and provides patients with effective choice and easier access to a second opinion. At the centre of the debate was the relationship between FDs and the relational continuity model. While some participants strongly viewed relational continuity with an FD as a source of rigidity, this would be perceived as less of a problem if the number of FDs was sufficient to provide choice.

From a policy perspective, the perceived importance of informational continuity is fundamental. Relational continuity appears to be less important if informational continuity works. Participants believed that GPs were less prone to errors if they knew their medical histories. This clearly challenges and opens new possibilities for the continuity model and for more flexible models of health care delivery. Satisfaction was also interrelated with views on organisational efficiency, management continuity, comprehensiveness and coordination. The relevance of these domains in our focus groups contrasts with the relative low importance found in (Grol *et al*., 2002).

Participants believed that reforms should not undermine the founding principles of NHS, mainly equity. However, the incomplete nature of the reforms and budget restrictions mean GP shortages limit patients’ choice, access to second opinion and raise concerns about inequity of PC provision. These results echoed Coulter, 2005 who states that individuals have aspirations as both patients and citizens regarding primary health care. FHUs are not universal and coverage is far from uniform across the country. This causes disparities in PC experiences described by the participants. Those assigned to an FHU describe better experiences with both curative and preventive care and higher satisfaction in any dimension of health care process. Despite similar reported levels of satisfaction, patients without an FD and those served by PHCUs believed they get inferior service. These groups reported difficulties in accessibility and doctor–patient relationship. These results are consistent with previous evidence. (Ferreira and Antunes, 2009; Ferreira *et al*., 2010; Ferreira and Raposo, 2015).

Despite the fact that reform of PC in Portugal is yet to be completed several years after it started, and despite the issues identified by focus group participants, the Portuguese remain supporters of publicly provided universal health care.

## References

[ref1] Administração Central do Sistema de Saúde (ACSS) (n.d.) Taxas Moderadoras - Atualização Dados. Retrieved April 2017 from http://www.acss.min-saude.pt/DownloadsePublica%C3%A7%C3%B5es/TabelaseImpressos/TaxasModeradoras/TaxasModeradorasAtualiza%C3%A7%C3%A3odedados/tabid/664/language/pt-PT/Default.aspx.

[ref2] Barros P , Simões J , Allin S and Mossialos E (2011) Portugal: Health system review. Health Systems in Transition 13, 1–156. 22222781

[ref3] Biscaia A and Heleno L (2017) A Reforma dos Cuidados de Saúde Primários em Portugal: portuguesa, moderna e inovadora. Ciência & Saúde Coletiva 22, 701–712. 28300980 10.1590/1413-81232017223.33152016

[ref4] Braun V and Clarke V (2006) Using thematic analysis in psychology. Qualitative Research in Psychology 3, 77–101.

[ref5] Chapman J , Zechel A , Carter Y and Abbot S (2004) Systematic review of recent innovations in service provision to improve access to primary care. British Journal of General Practice 54, 374–81. PMC1266174 15113523

[ref6] Chapple A , Campbell S , Rogers A and Roland M (2002) Users’ understanding of medical knowledge in general practice. Social Science & Medicine 54, 1215–1224. 11989958 10.1016/s0277-9536(01)00091-0

[ref7] Cheraghi-Sohi S , Bower P , Mead N , McDonald R , Whalley D and Roland M (2006) What are the key attributes of primary care for patients? Bulding a conceptual map of patients preferences. Health Expectations 9, 275–284. 16911142 10.1111/j.1369-7625.2006.00395.x PMC5060357

[ref8] Corbetta P (2003) Social research: theory, methods and techniques. London: Sage Publications. 10.4135/9781849209922.n11.

[ref9] Coulter A (2005) What do patients and the public want from primary care? BMJ 331, 1199–1201. 16293845 10.1136/bmj.331.7526.1199 PMC1285107

[ref10] Donabedian A (1988) The quality of care: how can it be assessed? JAMA 260, 1743–1748. 3045356 10.1001/jama.260.12.1743

[ref11] Ferreira P and Antunes P (2009) Monitorização da satisfação dos utilizadores das USF: sondagem às primeiras 146 USF. Coimbra: Centro de Estudos e Investigação em Saúde- Universidade de Coimbra (CEISUC).

[ref12] Ferreira P , Antunes P and Portugal S (2010) O valor dos cuidados primários: perspectiva dos utilizadores das USF-2009. s.l.: Centro de Estudos e Investigação em Saúde- Universidade de Coimbra (CEISUC).

[ref13] Ferreira P and Antunes P (2009) Sondagem às primeiras 146 USF. Coimbra: Centro de Estudos e Investigação em Saude- Universidade de Coimbra (CEISUC). Retrieved April 2017 from: http://www2.acss.min-saude.pt/Default.aspx?TabId=783&language=pt-PT.

[ref14] Ferreira P and Raposo V (2015) Monitorização da satisfação dos utilizadores das USF e de uma amostra de UCSP. Coimbra: Centro de Estudos e Investigação em Saúde da Universidade de Coimbra.

[ref15] Fung C , Elliott M , Hays R , Kahn K , Kanouse D , McGlynn E , Spranca M and Shekelle P (2005) Patients’ preferences for technical versus interppersonal quality when selecting a primary care physician. Quality of Care 40, 957–977. 10.1111/j.1475-6773.2005.00395.x PMC1361181 16033487

[ref16] Grol R , Wensing M , Mainz J , Ferreira P , Hearnshaw H , Hjortdahl P , Olesen F , Ribacke M , Spenser T and Szécsényi J (2002) Patients’ priorities with respect to general practice care: an international comparison. European Task Force on Patient Evaluations of General Practice (EUROPEP). International Journal for Quality in Health Care 14, 111–118. 10321388 10.1093/fampra/16.1.4

[ref17] Hjelmgren J and Anell A (2007) Population preferences and choice of primary care models: a discrete choice experiment in Sweden. Health Policy 83, 314–322. 17376559 10.1016/j.healthpol.2007.02.006

[ref18] INE (2009) Tipologia de áreas urbanas. Lisboa: Instituto Nacional de Estatística.

[ref19] INE (2017) Conta Satélite 2014 – 2016Pe. Retrieved May 2017 from https://www.ine.pt/ngt_server/attachfileu.jsp?look_parentBoui=297108579&att_display=n&att_download=y.

[ref20] Jung H , Baerveldt C , Olesen F , Grol R and Wensing M (2003) Patients characteristics as predictors of primary health care preferences: a systematic literature analysis. Health Expectations 6, 160–181. 12752744 10.1046/j.1369-6513.2003.00221.x PMC5060177

[ref21] Kitzinger J (1995) Qualitative research: introducing focus groups. BMJ 311, 299–302. 7633241 10.1136/bmj.311.7000.299 PMC2550365

[ref22] Krueger R and Casey M (2000) Focus groups: A practical guide for applied research. s.l.:Thousand Oaks, CA: Sage.

[ref23] MacDougall C and Fudge E (2001) Planning and recruiting the sample for focus groups and in-depth interviews. Qualitative Health Research 11, 117–126. 11147158 10.1177/104973201129118975

[ref24] Markham F , Diamond J and Hermansen C (1999) The use of conjoint analysis to study patients satisfaction. Evaluation & the Health Professions 22, 371–378. 10557865 10.1177/01632789922034365

[ref25] Mendes F , Mantovani M , Gemito M and Lopes M (2013) A satisfação dos utentes com os cuidados de saúde primários. Revista de Enfermagem ReferÃªncia vol.serIII, 9, 17–25.

[ref26] Ministério da Saúde (2017) Relatório Anual de Acesso aos Cuidados de Saúde nos Estabelecimentos do SNS e Entidades Convencionadas. Lisboa: Retrieved July 2018 from: https://www.sns.gov.pt/wp-content/uploads/2018/06/Relatorio_Acesso_SNS_2017_v.final_.pdf.

[ref27] Observatório Português de Políticas de Saúde (OPSS) (2013) Relatório de Primavera 2013 - duas faces da saúde. Retrieved May 2017 from: http://opss.pt/wp-content/uploads/2018/06/RelatorioPrimavera2013.pdf.

[ref28] Observatório Português dos Sistemas de Saúde (2012) Relatório da Primavera 2012 – Crise & Saúde. Retrieved May 2017 from: http://opss.pt/wp-content/uploads/2018/06/RelatorioPrimavera2012.pdf.

[ref29] Organisation for Economic Co-operation and Development - OECD (2017) OECD Health Data. OECD Health Statistics (database). Retrieved 2 July 2017 from http://www.oecd-ilibrary.org/social-issues-migration-health/data/oecd-health-statistics/system-of-health-accounts-health-expenditure-by-function_data-00349-en?citeformat%3Ddatabase.

[ref30] Santos O , Biscaia A , Antunes A , Craveiro I , Júnior A , Caldeira R and Charondière P (2007) Os Centros de Saúde em Portugal. Determinantes da satisfação com o funcionamento actual & prioridades da reforma. Uma abordagem qualitative. s.l.: Universidade Nova de Lisboa.

[ref31] Owen-Smith A and Coast J (2017) Understanding data collection: interviews, focus groups and observation. In Qualitative methods for health economics. s.l.: Rowman & Littlefield, 59–91.

[ref32] Richardson C and Rabiie F (2001) A Question of Access’ – an exploration of the factors influencing the health of young males aged 15–19 living in Corby and their use of health care. Health Education Journal 60, 3–6.

[ref33] Schers H , Webster S , van den Hoogen H , Avery A , Grol R and van den Bosch W (2002) Continuity of care in general pratice: a survey of patients views. British Journal of General Practice 52, 459–462. PMC1314320 12051209

[ref34] Scott A , Watson A and Ross S (2003) Eliciting preferences of the community for out of hours care provided by general praticioners: a stated preference discrete choice experiment. Social Science & Medicine 56, 803–814. 12560013 10.1016/s0277-9536(02)00079-5

[ref35] Simões J , Augusto G , Fronteira I and Hernández-Quevedo C (2017) Portugal - health systems review. Health Systems in Transition 19, 1–184. 28485714

[ref36] Tang K and Davis A (1995) Critical factors in the determination of focus group size. Family Practice 12, 474–475. 8826068 10.1093/fampra/12.4.474

[ref37] Turner D , Tarrant C , Windridge K , Bryan S , Boulton M , Freeman G and Baker R (2007) Do patients value continuity of care in general practice? An investigation using stated preference discrete choice experiments. Journal of Health Services Research and Policy 12, 132–137. 17716414 10.1258/135581907781543021

[ref38] Wensing M , Jung H , Mainz J , Olesen F and Grol R (1998a) A systematic review of the literature on patients priorities for general practice care. Part I: description of the research domain. Social Science and Medicine 47, 1573–1588. 9823053 10.1016/s0277-9536(98)00222-6

[ref39] Wensing M , Mainz J , Ferreira P , Hearnshaw H , Hjortdahl P , Olesen F , Reis S , Ribacke M , Szécsényi J and Grol R (1998b) General practice care and patients’ priorities in Europe: an international comparison. Health Policy 45, 175–186. 10338949 10.1016/s0168-8510(98)00040-2

[ref40] WHO/Europe (2010) Primary care evaluation tool. Retrieved on May 2017 from: http://www.euro.who.int/en/health-topics/Health-systems/primary-health-care/publications/2010/primary-care-evaluation-tool-pcet.

[ref41] Wong S , Watson D , Young E and Regan R (2008) What do people think is important about primary healthcare? Health Care Policy 3, 89–104. PMC2645139 19305771

